# The membrane associated NAC transcription factors ANAC060 and ANAC040 are functionally redundant in the inhibition of seed dormancy in *Arabidopsis thaliana*

**DOI:** 10.1093/jxb/erac232

**Published:** 2022-05-23

**Authors:** Shuang Song, Leo A J Willems, Ao Jiao, Tao Zhao, M Eric Schranz, Leónie Bentsink

**Affiliations:** Wageningen Seed Science Centre, Laboratory of Plant Physiology, Wageningen University, PB Wageningen, The Netherlands; Wageningen Seed Science Centre, Laboratory of Plant Physiology, Wageningen University, PB Wageningen, The Netherlands; Wageningen Seed Science Centre, Laboratory of Plant Physiology, Wageningen University, PB Wageningen, The Netherlands; Biosystematics Group, Wageningen University, PB Wageningen, The Netherlands; Biosystematics Group, Wageningen University, PB Wageningen, The Netherlands; Wageningen Seed Science Centre, Laboratory of Plant Physiology, Wageningen University, PB Wageningen, The Netherlands; John Innes Centre, UK

**Keywords:** *ANAC060*, *ANAC040*, *ANAC089*, coding sequence, functional redundancy, NAC transcription factor, promoter, seed dormancy, seed germination

## Abstract

The NAC family of transcription factors is involved in plant development and various biotic and abiotic stresses. The *Arabidopsis thaliana* ANAC genes *ANAC060, ANAC040, and ANAC089* are highly homologous based on protein and nucleotide sequence similarity. These three genes are predicted to be membrane bound transcription factors (MTFs) containing a conserved NAC domain, but divergent C-terminal regions. The *anac060* mutant shows increased dormancy when compared with the wild type. Mutations in *ANAC040* lead to higher seed germination under salt stress, and a premature stop codon in *ANAC089* Cvi allele results in seeds exhibiting insensitivity to high concentrations of fructose. Thus, these three homologous MTFs confer distinct functions, although all related to germination. To investigate whether the differences in function are caused by a differential spatial or temporal regulation, or by differences in the coding sequence (CDS), we performed swapping experiments in which the promoter and CDS of the three MTFs were exchanged. Seed dormancy and salt and fructose sensitivity analyses of transgenic swapping lines in mutant backgrounds showed that there is functional redundancy between *ANAC060* and *ANAC040*, but not between *ANAC060* and *ANAC089*.

## Teaser

The NAC transcription factors ANAC040, ANAC060 and ANAC089 are highly homologous, and promoter swapping experiments revealed that ANAC060 and ANAC040, but not ANAC089, are functionally redundant in the regulation of seed germination.

## Introduction

In *Arabidopsis thaliana*, 105 genes are predicted to encode NAC proteins (Ooka *et al.*, 2003). The NAC family is a composed of three transcription factors (TFs): *NAM* (*NO APICAL MERISTEM*), *Arabidopsis thaliana ACTIVATING FACTOR1, 2* (*ATAF1, 2*) and *CUC2* (*CUP-SHAPED COTYLEDON*; Souer *et al.*, 1996; Aida *et al.*, 1997). NAC TFs are described to contain a highly conserved N-terminal DNA binding domain also known as the NAC domain. Genome wide analysis revealed that more than 10% of the NAC TFs in Arabidopsis contain an α-helical transmembrane motif within their further varying C-terminal domain. These predicted membrane-associated domains determine the transcriptional activity and localization of the NAC proteins (Ernst *et al.*, 2004; Olsen, *et al.*, 2005; Hu *et al.*, 2006; S.Y. Kim *et al.*, 2007). NAC proteins are functionally diverse; they are involved in various developmental processes, including embryo and flower formation, organ separation, lateral root development and shoot apical meristem formation (Souer *et al.*, 1996; Aida *et al.*, 1997; Sablowski and Meyerowitz, 1998; Xie *et al.*, 2000; Takada *et al.*, 2001; Vroemen *et al.*, 2003; Weir *et al.*, 2004), as well as in biotic and abiotic stress defences, such as virus defence, wounding and microorganism defence, cold temperature sensitivity, drought responsiveness, and ABA sensitivity (Xie *et al.*, 1999; Ren *et al.*, 2000; Hegedus *et al.*, 2003; Fujita *et al.*, 2004; Tran *et al.*, 2004).

*ANAC060*, *ANAC040* and *ANAC089* belong to the same NAC sub-group, according to sequence similarity of their encoded proteins (Ooka *et al.*, 2003). All three proteins are predicted to be membrane-bound transcription factors (MTFs) since they contain a transmembrane domain (TMD; S.Y. Kim *et al.*, 2007; Li *et al.*, 2010). It has been shown that the full length ANAC060 protein, that contains the TMD, is associated with the nuclear membrane, whereas the truncated form lacking the TMD is localized in the nucleus (Li *et al.*, 2014). Similarly, full-length ANAC040 and ANAC089 proteins were mainly detected on plasma or endoplasmic reticulum membranes, and their truncated forms without the TMD, in the nucleus (S.G. Kim *et al.*, 2007; Yang *et al.*, 2014). For several Arabidopsis MTFs, it has been shown that the presence or absence of the TMD affects the plant phenotype (Kim *et al.*, 2010). *ANAC060* was described to affect sugar sensing. The Columbia (Col) allele of *ANAC060* encodes a truncated protein lacking the TMD, and its expression renders seedlings that are less sensitive to growth inhibition with high concentrations of sugar. In this process a role for abscisic acid (ABA) signalling via *ABSCISIC ACID INSENSITIVE 4* (*ABI4*) has been identified (Li *et al.*, 2014). Moreover, a T-DNA insertion in the *ANAC060* gene results in an increased seed dormancy phenotype when compared with Col-0 wild type (He, 2014).

ANAC040 is involved in several physiological processes. Overexpressing a truncated ANAC040 protein resulted in severe growth reduction and late flowering; the expression of *FLOWERING LOCUS T* was dramatically repressed in these lines (S.G. Kim *et al.*, 2007). Besides this, in the presence of high concentrations of salt, *anac040* mutant seeds could germinate to higher levels than wild type, and the germination of an over-expresser, containing the truncated protein lacking the TMD, was severely reduced (Kim *et al.*, 2008). Moreover, the gain-of-function mutant *anac040-1D* with increased expression of *ANAC040*, negatively regulated trichome formation by directly triggering the expression of *TRIPTYCHON* (*TRY*) and *TRICHOMELESS1* (*TCL1*), two genes that repress the formation of trichomes. Notably, the observed similar trichome phenotype of the truncated and full length ANAC040 protein in transgenic lines imply that in both transgenic lines the ANAC040 protein ends up in the nucleus. However, how ANAC040 re-localizes to the nucleus remains elusive (Schnittger *et al.*, 1998; Wang *et al.*, 2007; Tian *et al.*, 2017).

*ANAC089* is elevated by endoplasmic reticulum (ER) stress and the truncated form of *ANAC089* lacking the TMD activates programmed cell death; this activity is controlled by bZIP28 and bZIP60 which are two known MTFs playing crucial roles in regulating cell viability during plant ER stress (Yang *et al.*, 2014). Recently, it was reported that the translocation of ANAC089 to the nucleus is directed by changes in cellular redox status after treatment with nitric oxide (NO) scavengers and redox-related compounds (Albertos *et al.*, 2021). This study revealed ANAC089 as a master regulator modulating redox homeostasis and NO levels during seed germination and abiotic stress. Moreover, the localization of *ANAC089* is also determined by natural genetic variation, as it was described for *ANAC060*. The Cape Verde Islands (Cvi) allele of *ANAC089* suppresses fructose signalling, due to a premature stop codon; this protein lacks the TMD, resulting in nuclear localization. In contrast, the L*er* allele, that contains the TMD, is localized in the cytoplasm and sensitive to fructose (Li *et al.*, 2011).

To investigate possible redundancy between these highly homologous NAC transcription factors, Tian *et al.* (2017) compared the trichome phenotype in rosette leaves of the *anac060 anac040* double mutants to their single mutants. No differences in trichome formation on rosette leaves was found, when comparing *anac040*, *anac060*, and *anac060anac040*. However, the double mutant did show more branched trichomes on the stems, which suggested a possible functional redundancy. It is well known that the function of eukaryotic genes is determined by its distinct functional constituents, for example, the enhancer/silencer, the promoter region, and the coding sequence (CDS). Among these, the promoter and the CDS are the two central functional domains. The promoter region regulates the timing and pattern of gene expression. The CDS encodes the protein which is responsible for the phenotype (Polyak and Meyerson, 2003).

Here, we have performed promoter and CDS swapping experiments to disclose the functional redundancy and distinction of functions between *ANAC060*, *ANAC040*, and *ANAC089*. These experiments revealed that there is functional redundancy between *ANAC060* and *ANAC040*; the different phenotypes of the native genes are likely the result of their distinct expression patterns. We did not detect functional overlap between *ANAC060* and *ANAC089*.

## Materials and methods

### Plant materials and growth conditions

Seeds of the *Arabidopsis thaliana* accession Columbia (Col-0), *anac060-1* mutant (SALK_127838C), *anac060-2* mutant (SALK-012554C), *anac040* mutant [SM_3.16309; alias *anac040-1* mutant (SM_3.16309; S.G. Kim *et al.*, 2007)] and *anac040-2* (SM_3_16309; Tian *et al.*, 2017) were obtained from the Nottingham Arabidopsis Stock Centre, UK. The mutant *anac089* (Gt19255) in the Landsberg *erecta* (L*er*) genetic background was obtained from Sheng Teng, Institute of Plant Physiology and Ecology, Shanghai Institute of Biological Sciences (Li *et al.*, 2011).

***Seed production***

Seeds were sown on water-imbibed white filter paper in transparent petri dishes, and then placed in a dark room at 4 °C for cold stratification. After 3 d, seeds were transferred to a growth chamber at 22 °C with continuous light for another day before planting. Germinated seeds with radical protrusion were grown on 4 × 4 cm Rockwool blocks in a climate room at 20 °C/18 °C (day/night) under a 16 h artificial light (150 μmol m^-2^ s^-1^) photoperiod and 70% relative humidity. Plants were watered with a standard nutrient solution three times per week. Seeds were harvested when the majority of the siliques had turned yellow. The seeds of each genotype were harvested in three seed bulks of four plants, each bulk serving as a biological replicate.

### Seed germination experiments

***Seed dormancy comparisons***

Seeds (50–100) were sown in plastic trays (15 × 21 cm) on two layers of blue paper with 48 ml demineralized water, using a plastic mask to accurately position the seed samples. Following this, the trays were piled and wrapped into transparent plastic bags. The trays were placed into an incubator at 22 °C, 138 µmol m^-2^ s^-1^ continuous light. Photographs to assess the germination were taken twice a day during a 7 d cycle. Germination was scored using the Germinator package (Joosen *et al.*, 2010). Dormancy level was evaluated by the days of seed dry storage required to reach 50% of germination (DSDS50). The method to determine DSDS50 has been described previously (Bentsink *et al.*, 2010).

***Salt and fructose treatments***

Around 50 surface sterilized seeds were plated on sterilized 1.2 % agar, half-strength Murashige and Skoog (MS) medium containing either 150, 200, 250 mM mannitol or NaCl, 6.5% sorbitol or 5.5, 6.0, and 6.5% fructose. For all conditions the pH was set to 5.8. Seeds were stratified at 4 °C in the dark for 2 d, and then exposed to continuous light for 5-7 d at 22 °C. in The calibration curves for the different salt and fructose concentrations are shown in Supplementary Fig. S1 and Fig. S2, respectively. For salt treatments: germination percentage was calculated on a daily base. Seeds with a radicle occurrence were categorized to be germinated (Kim *et al.*, 2008). For fructose treatment, fructose sensitivity phenotype was evaluated by counting the percentage of green seedlings with expanded cotyledons (Li *et al.*, 2011).

### DNA extraction from seeds

Around 200 seeds and two small bullets were placed in 1.5 ml tubes, and seeds were ground for 1 min, at 30 Hz using a mixer mill (MM 400, RETSCH, Belgium); following grinding, 250 µl extraction buffer (2 M NaCl, 200 mM Tris-HCl pH 8, 70 mM EDTA, 20 mM Na_2_S_2_O_5_) was added to each sample and ground again, as specified above. Samples were incubated at 60 °C for 1 h. After that, samples were centrifuged for 10 min at maximum speed, 75 µl clean supernatant was taken from each tube into a new tube, 75 µl iso-propanol and 30 µl of 10 M NH_4_AC were added and mixed well in each tube. All samples were kept for 15 min at ~20 °C for precipitation; after that they were centrifuged for 20 min at maximum speed. The supernatant was discarded and the DNA pellet was washed in 70 µl of 70% ethanol, after which the samples were centrifuged for 5 min at maximum speed. The ethanol was removed using a pipette, and the DNA pellet was dried for 10 min. Finally, the pellet was dissolved in 30 µl of Milli-Q water (Cheung *et al.*, 1993).

### PCR conditions and gel electrophoresis

PCR was operated in a 25 µl total volume including around 100 ng DNA, 1.25 µl of 10 µM of forward and reverse primers (for primer sequences see Supplementary Table S1), and 12.5 µl VYO HIFI mix (PB10.43, SOPACHEM, The Netherlands). The cycling programme was as follows: first denaturation at 94 °C for 3 min in one cycle, followed by second denaturation at 94 °C for 10 s. Annealing temperature ranged from 59–64 °C (depending on the primers) for 30 s, and a 45 s extension at 72 °C; this cycle was repeated 35 times. The final extension was at 72 °C for 5 min. The PCR products were checked by electrophoresis in 1% agarose gel.

### Sequence comparisons

#### Unrooted phylogenetic tree

*ANAC060* was analysed by using a plant membrane protein database (Schwacke *et al.*, 2003), and 15 high homology genes were collected. An unrooted phylogenetic tree for these 15 sequences was built by using MEGA 7.0 (Kumar *et al.*, 2016) and the UPGMA method (Sneath and Sokal, 1973). The units of the branch lengths in the sub-group are comparable to the evolutionary distances used to interpret the phylogenetic tree. Poisson correction was used to compute the evolutionary distances (Zuckerkandl and Pauling, 1965).

#### Proteins sequence alignments

ANAC060, ANAC040, and ANAC089 sequences from Columbia are derived from The Arabidopsis Information Resource (TAIR, www.arabidopsis.org). The NAC domain was predicted by the plant transcription factor database (Schwacke *et al.*, 2003) and the transmembrane domain was analysed by TMHMM Server v.2.0 (Sonnhammer *et al.*, 1998; Krogh *et al.*, 2001).

### Synteny network construction and phylogenetic reconstruction

The synteny network of the NAC gene family was constructed by extracting the syntenic relations of all the NAC genes from the synteny network database of 107 plant genomes (Zhao and Schranz, 2019). To do this, the NAC gene IDs identified from the 107 plant genomes using HMMER3 (Mistry *et al.*, 2013) was used to query the network database. The edge list of the NAC sub-network was clustered and visualized in Gephi ­(Bastian *et al.*, 2009). We located and highlighted the synteny cluster containing *ANAC060*, *ANAC040*, and *ANAC089*. A closely inter-connected synteny cluster indicates a shared genomic context. The phylogenetic tree was then reconstructed for the nodes/genes in this cluster. Multiple sequence alignments were performed using MAFFT (version 7.187). Alignment trimming were conducted by trimAl (Capella-Gutiérrez *et al.*, 2009). Maximum-likelihood analyses were conducted using IQ-TREE (Nguyen *et al.*, 2015). We used the ‘JTT+R’ model for protein sequence alignment, with 1000 bootstrap replicates (-bb 1000).

### Construction of expression vectors by Gateway cloning

Gateway cloning was performed according to the manual Multisite Gateway® Pro: Using Gateway® Technology. This method allows to simultaneously clone multiple DNA fragments. All primers used for cloning are listed in Supplementary Table S1.

pCol-0_*ANAC040*::Col-0_*ANAC040ΔC* construct: The native *ANAC040* promoter (1610 bps) was cloned from accession Col-*0* (pCol-0_*ANAC040*) and the genomic sequence containing the coding region of *ANAC040* with an added stop codon was also cloned from wild type Col-0 (Col-0_*ANAC040ΔC*; S.G. Kim *et al.*, 2007). The promoter and the genomic sequence were amplified using the VYO HIFI mix and cloned into Gateway entry vectors pDONR 221 + P1P5 and pDONR 221 + P5P2 (Invitrogen Life Technologies), respectively. Following this, the two entry vectors were cloned into the destination vector 428pKGW red seed + R1R2 (Jan Verver, Laboratory of Molecular Biology, WUR, The Netherlands; Invitrogen Life Technologies) which allows transformant selection based on fluorescence.

pCol-0_*ANAC089*::Cvi_*ANANC089* construct: The native *ANAC089* promoter (1796 bp) was cloned from ecotype Col-0 (pCol-0_*ANAC089*) and the genomic sequence containing the coding region of *ANAC089* was cloned from accession Cvi (Cvi_ *ANAC089*; Li *et al.*, 2011). The construct was generated as explained for pCol-0_*ANAC040*::Col-0_*ANAC040ΔC* construct.

pCol-*0*_ *ANAC060*::L*er*_*ANAC060* construct: The native *ANAC060* promoter (1746 bp) was cloned from PMD18_Col-*0* (Li *et al.*, 2014). The genomic sequence containing the coding region of *ANAC060* was cloned from L*er*. The construct was generated as described for pCol-*0*_*ANAC040*::Col-*0*_*ANAC040ΔC* construct.

### Swapping experiment

Recombined constructs in which the promoters and the genomic sequences containing the coding regions of *ANAC060*, *ANAC040*, *ANAC089* were swapped, were the same as that described above, as shown in Supplementary Fig. S3.

### Transformation of *Arabidopsis thaliana*

Agrobacterium cells containing the constructs were centrifuged for 15 min at 4000 rpm at ~20 °C in 250 ml tubes; the supernatant was poured off, and the pellet was resuspended in infiltration medium (half-strength MS medium + vitamins, 5% sucrose) to an OD_600_ =1. Prior to be used, 0.03% Silwet L-77 was added to the mixture. Following this, the flowering plants were dipped into the culture for 15 s. The plants were placed in plastic bags in a horizontal position overnight. After the plastic bags were opened, the plants were grown in an upright position until seeds were to be harvested.

### RNA isolation and characterization

Total RNA was extracted according to the hot borate protocol described by Maia *et al.* (2011). In brief, 3–3.5 mg of seeds for each treatment were homogenized and mixed with 800 µl of extraction buffer containing dithiothreitol (DTT) and PVP40 which had been heated to 80 °C. Proteinase K was added and incubated for 15 min at 42 °C. After adding 2 M KCl, the samples were incubated on ice for 30 min and centrifuged. Ice-cold 8 M LiCl was added to the supernatant and the tubes were incubated overnight on ice. After centrifugation, the pellets were washed with ice-cold 2 M LiCl and centrifuged for 10 min. The pellets were resuspended in 80 µl DEPC milli-Q water. The samples were phenol:chloroform extracted, DNase treated and further purified. RNA quality and concentration were assessed by agarose gel electrophoresis and Nanodrop ND1000 spectrophotometry.

### cDNA synthesis and qPCR analysis

iScript cDNA synthesis kit (Bio Rad, The Netherlands) was used for making cDNAs in this project, and 1 μg total RNA was used from each sample. cDNA samples were diluted 10-fold. qPCR was performed according to the manufacturer’s guidelines (Eurogentec, Belgium). The master mix was 10 μl for each reaction: 2.5 μl cDNA, 5 μl SYBR green (Sopachem BV, The Netherlands, VYBA01-31), 0.5 μl primer mixtures, 2 μl MQ water. CFX Bio Rad was used to generate threshold cycle value for each reaction.

qPCR primers and analyses: *ANAC040*, forward primer: AGGATGCATTAGTGGTGTGC, reverse primer: TTGTCCTCCTTCTCCAAACC; *ANAC060*, forward primer: AGCCTTGGGATTTACCTGA, reverse primer: TTGGTTGCTCTTCTGTTCTGT. Two reference genes, *At4G12590* and *At4G23270,* were designed based on a study by (Dekkers *et al.*, 2012). The Ct value of the gene of interest was normalized to the average Ct value of the two reference genes according to the equation: ∆Ct = Ct (gene of interest) – average Ct (reference genes). The ∆Ct of every genotype was compared with the average ∆Ct of the control line (Col-0), this was referred as the ∆∆Ct value with the equation: ∆∆Ct = ∆Ct (gene of interest in each genotype) – average ∆Ct (gene of interest in Col-0). Finally, expression was expressed as fold change of the ∆∆Ct value = 2^–(∆∆Ct)^.

### Motif analyses

Predictive promoter sequences of three genes were analysed using the PlantCARE database (plant *cis*-acting regulatory elements; Lescot *et al.*, 2002), and qualitative motifs of three single genes are presented and described in Supplementary Table S2.

## Results

### The evolutionary relationships of *ANAC060*, *ANAC040* and *ANAC089*

**Fig. 1.**
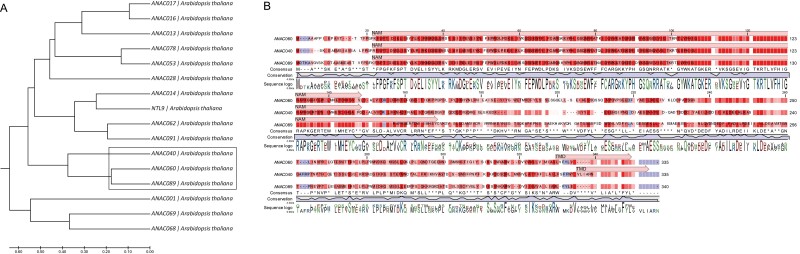
Sequence comparisons of *ANAC060, ANAC040* and *ANAC089*. (A) The unrooted phylogenetic tree was built by using MEGA 7.0 (Kumar *et al.*, 2016) and the UPGMA method (Sneath and Sokal, 1973). The units of the branch lengths in the sub-group are comparable to the evolutionary distances used to interpret the phylogenetic tree. Poisson correction was used to compute the evolutionary distances (Zuckerkandl and Pauling, 1965). The tree scale is shown below the tree. (B) Protein sequences alignments for ANAC060, ANAC040, and ANAC089. Sequences are derived from the Columbia genome (TAIR). The NAC domain was predicted using the plant transcription factor database (Schwacke *et al.*, 2003) and the transmembrane domain was analysed by TMHMM Server v.2.0 (Sonnhammer *et al.*,1998; Krogh *et al.*, 2001).

**Fig. 2.**
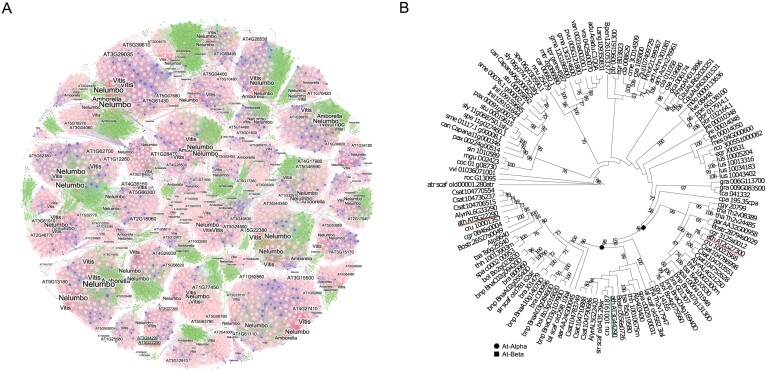
Evolutionary analysis of *NAC* genes. (A) Synteny network of *NAC* genes in 107 angiosperm genomes. The size of each node corresponds to the number of edges it has (node degree). Communities were labelled by the sub-families/sub-family involved. The different colours in each cluster represent genes belonging to rosids (light pink), monocots (green), asterids (purple) (Zhao *et al*., 2017, 2019). *ANAC060*, *ANAC040* and *ANAC089* are underlined with the colours moss, salmon, and mocha respectively. (B) Phylogenetic analysis of *ANAC060*, *ANAC040*, and *ANAC089*. *ANAC040* (*AT2G27300*, underlined with salmon) was retained from the older At-Beta whole genome duplication event. The species used to create this tree are listed in Supplementary Table S3. *ANAC060* (*AT3G44290*) and *ANAC089* (*AT5G22290*) are indicated by moss and mocha lines correspondingly. The black round and square nodes stand for At-Alpha and At-Beta polyploid events respectively. The bootstrap range is displayed from 58 onwards.

Protein sequence comparisons were performed to identify genes with high homology to *ANAC060* in Arabidopsis. The 15 most homologous genes were selected based on an earlier comparison of the protein sequences of ANAC transcription factors (Schwacke *et al.*, 2003). ANAC040 (41% identity) and ANAC089 (64% identity) were found to be the two most homologous proteins to ANAC060 based on sequence similarity (Pearcon and Lipman, 1988; Pearson, 2000; Fig. 1A). The homology is especially high at the N terminal part of the protein that contains the NAC domains (NAM) from 21-146 amino acids (Fig. 1B). To investigate the evolutionary history of the three *NAC* genes, we extracted the whole synteny network of the NAC gene family from the entire synteny network database constructed from 107 plant genomes (Zhao and Schranz, 2019). Interestingly, *ANAC060*, *ANAC040* and *ANAC089* were located in the same synteny cluster which indicated a common genomic origin, shared with other eudicots, but lacking monocots (Fig. 2A). The constructed phylogenetic tree of this cluster included many Brassicaceae sequences and sequences from *Cleome gynandra* (cgy) and *Tarenaya hassleriana* (tha), both of which belong to the Cleomaceae family, which is the sister lineage to Brassicaceae (Bowers *et al.*, 2003). Using this information, we propose the following duplication history and evolution of the three genes. *ANAC040* and *ANAC060/ANAC0890* represent duplicates derived from the older At-Beta whole genome duplication event and *ANAC060* and *ANAC0890* were duplicated from At-Alpha whole genome duplication event (shared only by *Brassicaceae* species; Fig. 2B).

### The *ANAC040* coding sequence rescues the *anac060* dormancy phenotype

**Fig. 3.**
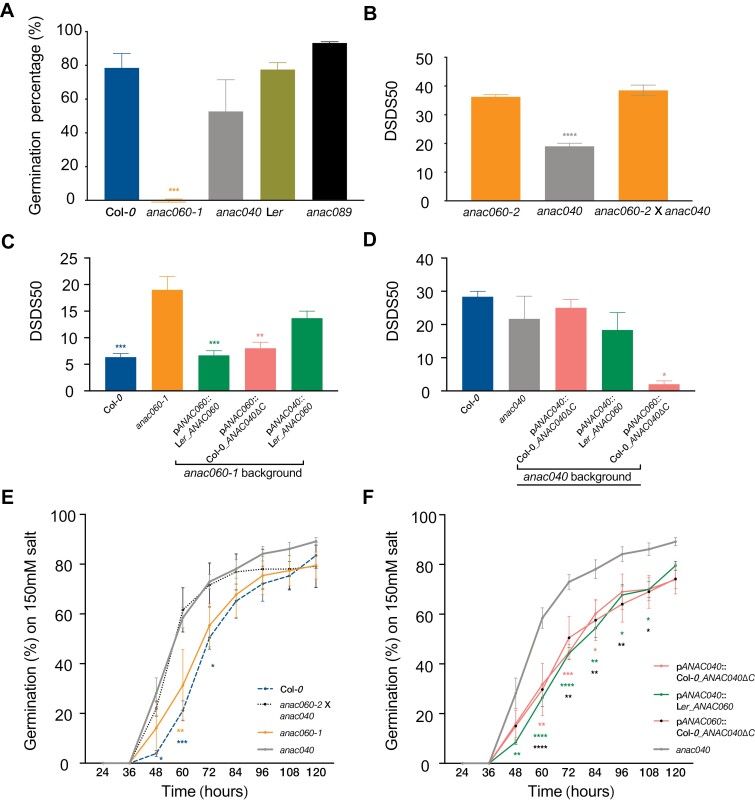
Dormancy and salt phenotypes of swapped transgenic lines. (A) Dormancy phenotype of the *anac060-1*, *anac040*, and *anac089* mutants and their respective wild types. Dormancy is measured as germination percentage one week after seed harvest. The lower the germination percentage the higher the dormancy level. (B) Dormancy phenotype of *anac060-2*, *anac-40*, and the *anac060-2 anac040* double mutant. Seed dormancy is displayed as DSDS50 (days of seed dry storage required to reach 50% germination) levels. The higher the DSDS50 value the higher the dormancy level. (C) Dormancy phenotype of transgenic lines in the *anac060-1* genetic background. (D) Dormancy phenotype of transgenic lines in the *anac040* background at 25 °C. (E, F) Salt sensitivity of mutants and transgenic lines in the *anac040* genetic background. Bars indicate the mean value of three replicates and the SE. Statistical significances were calculated using one-way ANOVA in (A-D), and two-way ANOVA in (E) and (F). Asterisks indicate significant differences with the respective backgrounds *anac060-1* or *anac040* (for A-D) and significant differences with *anac040* (for E and F; *P≤0.05, **P≤0.01, ***P≤0.001 and ****P≤0.0001).

To identify functional redundancy between *ANAC060* and its homologs *ANAC040* and *ANAC089,* the mutants were investigated for their dormancy phenotypes. Seeds of *anac060-1* and *anac060-2* both showed deeper primary dormancy than wild type Col-0. The primary dormancy levels of *anac040* and *anac089* did not significantly differ from those of their respective wild types Col-0 and L*er* with 1 week after-ripening (Fig. 3A). Moreover, the dormancy phenotype of the *anac060-2 anac040* double mutant did not differ significantly from the single *anac060* mutant, indicating that *ANAC060* is epistatic over *ANAC040* in dormancy regulation (Fig. 3B). The double mutant between *anac060* and *anac089* was not constructed because of the different genetic backgrounds of the available mutants, i.e. Col-0 and L*er*, respectively. Phenotypic analyses of such a cross would be complicated due to genetic segregation of seed dormancy loci that are present in L*er* and Col-0 (van der Schaar *et al.*, 1997).

To investigate whether the promoter or CDS could lead to functional redundancy, promoters and CDS swapping experiments were performed. For the swapping experiments, all promoters were cloned from Col-0. The active CDSs, thus the alleles that result in a lack of the TMD, were selected and cloned from *ANAC060*, *ANAC40*, and *ANAC089*. Seeds of *anac060-1*, Col-0, and transgenic lines containing the p*ANAC060*::Col-*0*_*ANAC040ΔC* construct (ΔC refers to the allele that lacks the TMD), p*ANAC040*::L*er*_*ANAC060* and p*ANAC060*::L*er*_*ANAC060* in *anac060-1* background, were examined for their dormancy level (measured as DSDS50; days of seed dry storage required to reach 50% of germination). The transgenic lines containing the p*ANAC060*::L*er*_*ANAC060* and p*ANAC060*::*Col-0*_*ANAC040ΔC* constructs both complemented the *anac060-1* dormancy phenotype. This was not the case for the recombinant lines expressing the p*ANAC040*::L*er*_*ANAC060* construct (Fig. 3C).

To further investigate the redundancy between *ANAC060* and *ANAC040*, the same constructs were transformed into the *anac40* mutant background. As described above, the Col-0_*ANAC040* contains a TMD and there is no dormancy difference between *anac040* and Col-0 in our standard dormancy testing conditions (22 °C, continuous light). In order to be able to identify small differences in seed dormancy, germination experiments were also performed at 25 °C. During seed storage (after-ripening) the germination window widens, meaning that seeds can germinate better in less optimal conditions, e.g. higher temperatures (Alvarado *et al.*, 2002). This phenomenon allowed us to investigate whether p*ANAC060*::Col-0_*ANAC040ΔC* could release seed dormancy in the *anac40* mutant background. There was no difference in dormancy between *anac040*, Col-0 and transgenic line p*ANAC040*::Col-0_*ANAC040ΔC* in the *anac040* background. However, the transgenic line p*ANAC060*::Col-0_*ANAC040ΔC* was less dormant than its background line *anac040*, showing again that ANAC040, when present in the nucleus, can overcome seed dormancy (Fig. 3D).

Moreover, the single and double mutants and the transgenic seeds were also tested for salt sensitivity, according to the study by Kim *et al.* (2008). A concentration of 150 mM NaCl was most discriminative for *anac040* and Col-0 (Supplementary Fig. S1). *anac040* and the double mutant *anac060-1 anac040* were resistant to 150 mM salt, whereas Col-0 and *anac060-1* were not. These results indicated that *ANAC040* is epistatic over *ANAC060* in regulating salt sensitivity (Fig. 3E). The p*ANAC040*::Col-0_*ANAC040ΔC* construct complemented the mutant *anac040* phenotype. The transgenic lines containing the swapped constructs p*ANAC060*::Col-0_*ANAC040ΔC* and p*ANAC040*::L*er*_*ANAC060* also displayed significant lower germination percentages than *anac040* in the presence of 150 mM salt (Fig. 3F).

**Fig. 4.**
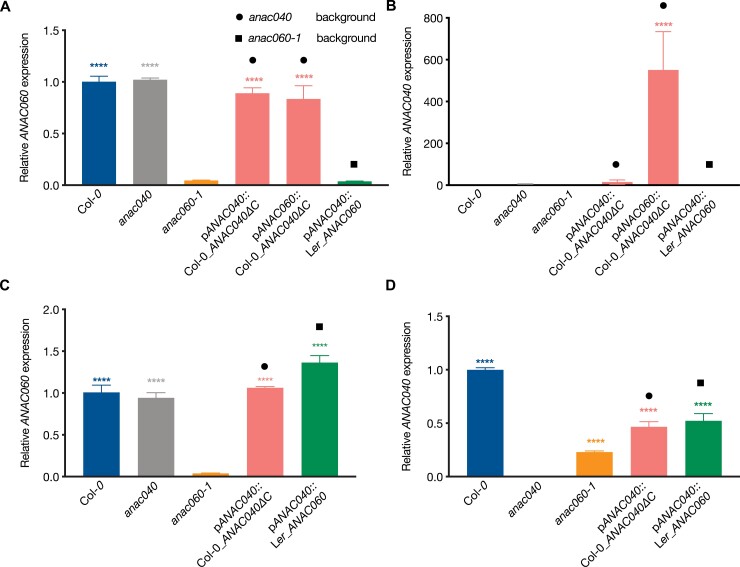
Expression of *ANAC060* and *ANAC040* in respective lines determined by RT–qPCR. (A, B) Expression level of *ANAC060* and *ANAC040* in dry seeds. (C, D) Expression level of *ANAC060* and *ANAC040* in 3 d cold-imbibed seeds. Expression in Col-0 was used as a control and its value was set as 1. Solid rounds and squares indicate the respective mutant backgrounds *anac040* and *anac060-1*. The expression level of all other lines was relative to 1. Statistical significances were calculated using one-way ANOVA. Significance differences were compared between each transgenic line and its background mutant. Bars indicate the mean value of three replicates and the SE. Asterisks indicate significant differences between the transgenic lines and their respective mutant backgrounds (***P≤0.001, ****P≤0.0001).

The results described above showed that there is functional redundancy between the *ANAC040* and *ANAC060* CDS, however there were distinct phenotypes under the different test conditions. For example, the transgenic line containing the p*ANAC040*::Col-0_*ANAC040ΔC* construct did not induce seed dormancy in the *anac040* mutant background, whereas the p*ANAC060*::Col-0_*ANAC040ΔC* construct did (Fig. 3D). Expression analyses were performed in dry seeds to investigate if these phenotypes could be explained by the expression level of the transgenes, thus the activity of the promoters. *ANAC060* expression was down-regulated in the *anac060* mutant dry seeds and showed a wild type level of expression in Col-0 and the *anac040* mutant (Fig. 4A). *ANAC040* was not expressed in dry seeds but was significantly induced (*P*≤0.0001) under the *ANAC060* promoter in the *anac040* mutant background (Fig. 4B). As reported by Kim *et al.* (2008), *ANAC040* was strongly induced in 3 d cold-imbibed seeds. We confirmed the promoter activity of p*ANAC040* after 3 d of cold stratification by investigating *ANAC060* expression in the p*ANAC04*0::L*er_ANAC060* transgenic line (*anac060-1* background; Fig. 4C). Also, *ANAC040* expression was induced in Col-0 and, although to a slightly lower level, in the transgenic lines containing the *ANAC040* promoter (Fig. 4D). Moreover, *ANAC040* expression was significantly reduced in the *anac060-1* mutant (*P*≤0.0001); however this was also partly rescued in the p*ANAC040*::L*er_ANAC060* transgenic line (*anac060-1* background; Fig. 4D).

**Fig. 5.**
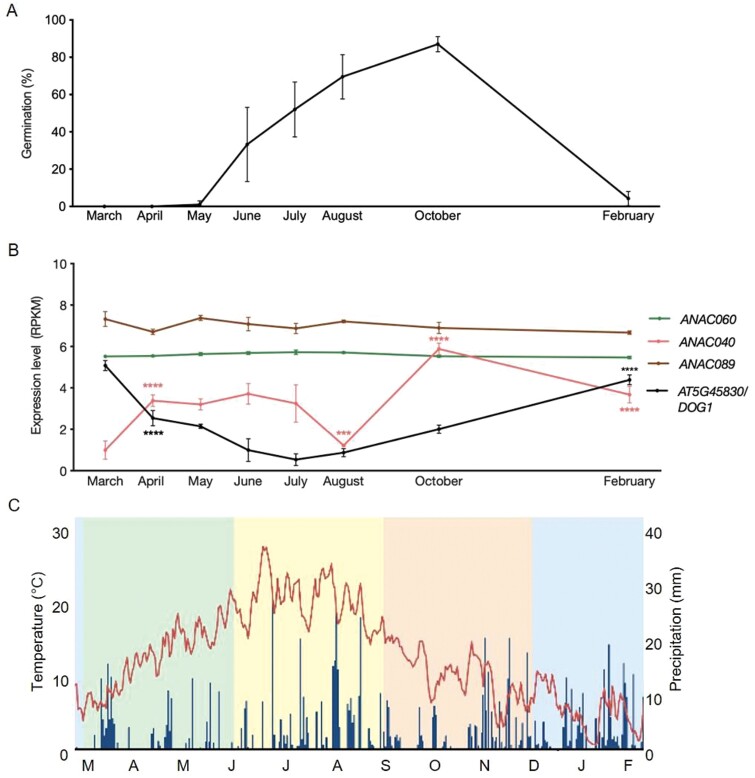
Expression levels of *ANAC060*, *ANAC040*, *ANAC089,* and *DOG1* (*AT5G45830*) in dormancy cycling under soil. (A) Germination percentage throughout the seasons. Landsberg *erecta* seeds were collected from the field and germinated under laboratory conditions. (B) Relative expression levels of *ANAC040*, *ANAC060*, *ANAC089* and *DELAY OF GERMINATION 1* (*DOG1*) during dormancy cycling. RPKM levels are shown. (C) Temperature and moisture content at 5 cm depth in the soil. Red lines indicate field temperature obtained from buried sensors. The data presented in (A-C) are derived from Buijs *et al.* (2020). Two-way ANOVA was performed to analyse the data, every comparison was between the adjacent previous month, asterisks indicate significant differences (***P≤0.001, ****P≤0.0001). In (A) and (B), the mean value of four replicates and their SE are presented.

The redundancy between *ANAC060* and *ANAC040* was also indicated by the double mutant analyses (Fig. 3B). This epistatic behaviour of *ANAC060* is likely explained by the lack of *ANAC040* expression in dry seeds (Fig. 4B). When comparing the germination percentage of *anac060-1 anac040* double mutant to the single *anac040* and *anac060-1* mutants in the presence of 150 mM salt, only a significant difference between the single mutants was confirmed (Fig. 3E). *ANAC040* is induced during seed imbibition especially in response to cold (Fig. 4D); it can overcome seed dormancy under the control of the promoter of *ANAC060* (Fig. 3D). Based on these findings a role for *ANAC040* in the regulation of dormancy cycling was predicted. Secondary dormancy can be induced when the conditions are not optimal for seeds to germinate (Baskin and Baskin, 1998; Buijs, 2020). To test this hypothesis, we made use of public expression data describing a dormancy cycling experiment that was performed by following seed germination behaviour and transcriptional changes during one year of seed burial (Buijs *et al.*, 2020). From March to May, buried seeds remained deep dormancy and re-gained germination capacity in June, after which the germination ability gradually increased to 100% in October. During winter seeds became dormant again resulting in a reduced germination capability (20%) in February (Fig. 5A; Buijs *et al.*, 2020). The expression of *ANAC040*, *ANAC060* and *ANAC089* during dormancy cycling was investigated and compared with that of *DELAY OF GERMINATION 1* (*DOG1)*, a gene that has been reported to show differential expression during dormancy cycling (Footitt *et al.*, 2015; Murphey *et al.*, 2015). The higher relative expression of *ANAC040* in the non-dormant phase of dormancy cycling is in agreement with our earlier findings that *ANAC040* overcomes seed dormancy (Figs 3D, 5B). Its peak in expression in October might be explained by the sharp drop in temperature, since we know that low temperatures induced *ANAC040* expression (Figs 4D, 5C.

### Motif analyses in promotors of *ANAC060* and *ANAC040*

Motif analyses on the promoters of *ANAC060* (5249 bp) and *ANAC040* (1998 bp) were performed to determine whether these motifs could explain the differential expression of *ANAC060* and *ANAC040* (Lescot *et al.*, 2002). In total 16 and 14 motifs were identified in the *ANAC060* and *ANAC040* promoters, respectively; 12 of these regulatory motifs were present in both promoters. Four motifs are specific for *ANAC060* and two are specific for *ANAC040* (Table 1). Among these motifs are the ABA-responsive element (ABRE), MYB, G-box, W-box, and GT1-motif, which have been associated with regulation of Arabidopsis seed development (Belmonte *et al.*, 2013; Yamasaki *et al.*, 2017).

**Table 1.** Motifs identified in the promotors of *ANAC060* and *ANAC040*.

	Number	Elements	Sequence	Function
Motifs in p*ANAC040* and p*ANAC060*	12	MYB/MYB-like sequence/ Myb	TAACCA/ TAACTG	Responding to hormones during seed dormancy and gemination (Abe *et al.*, 1997)
		activation sequence-1 (as-1)	TGACG	Salicylic acid (SA)- and auxin-responsive element responding to reactive oxygen species (Garretón *et al.*, 2002)
		AT~TATA-box TATA TATA-box	TATATA/ TATAAAAT/ TATA	Core promoter element around -30 of transcription start
		ABRE	TACGGTC	*Cis*-acting element involved in the abscisic acid, high salinity, drought responsiveness (Maruyama *et al.*, 2012)
		CAAT-box	CCAAT	Common *cis*-acting element in promoter and enhancer regions
		G-box	CACGTG	*Cis*-acting regulatory element involved in light responsiveness; Regulating seed specific expression (Ouwerkerk and Memelink, 1999)
		GT1-motif	GGTTAA	Light responsive element
		TCT-motif	TCTTAC	Part of a light responsive element (Lam and Chua, 1990)
Motifs in p*ANAC060*	4	W box	TTGACC	Binding site for WRKY transcription factors (Rushton *et al.*, 2010)
		Activator Protein 1 (AP-1)	TGAGTTAG	Responding to oxidative stress in different organisms (Karin *et al.*, 1997; Shaulian and Karin *et al*., 2001; Lev *et al.*, 2005; Scandalios, 2005)
		MYC	CATGTG	Function in regulating dehydration-inducible gene (Tran *et al.*, 2004)
		CAT-box	GCCACT	*Cis*-acting regulatory element related to meristem expression
Motifs in p*ANAC040*	2	STRE	AGGGG	Heat shock elements (Ruis and Schüller, 1995; Martínez-Pastor *et al.*, 1996; Estruch, 2000; Guo *et al.*, 2008)
		GA-motif	ATAGATAA	Part of a light responsive element

### There is no functional redundancy between *ANAC060* and *ANAC089*

**Fig. 6.**
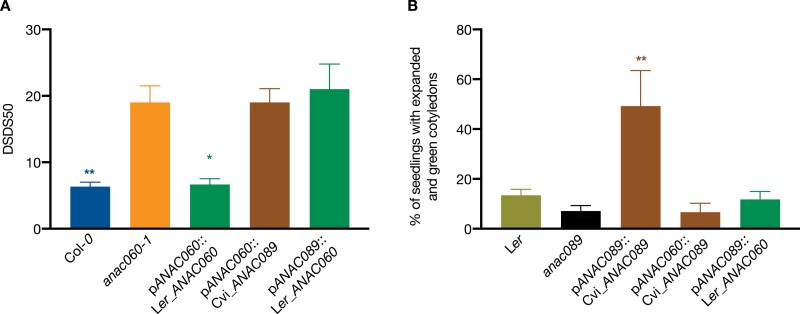
No functional redundancy between *ANAC060* and *ANAC089*. (A) Dormancy of *ANAC060* and *ANAC089* swapping lines. Seed dormancy is shown as DSDS50 (days of seed dry storage required to reach 50% germination) levels. (B) Fructose sensitivity of transgenic lines. Seeds were sown on half-strength MS plates and ones contain 6% fructose plates. Statistical significance was calculated using one-way ANOVA. Bars indicate the mean value of three replicates and the SE. Asterisks indicate significant differences compared with the respective single mutants (*P≤0.05, **P≤0.01).

To test whether *ANAC089* can rescue the dormancy phenotype of *anac060-1,* seed dormancy levels of *anac060-1*, Col-0 and transgenic plants containing the p*ANAC060*::Cvi_*ANAC089* and p*ANAC089*::L*er*_*ANAC060* constructs in *anac060-1* background were measured. None of these swapped transformants complemented the *anac060-1* dormancy phenotype (Fig. 6A). Similarly, we tested whether *ANAC060* could complement the *anac089* fructose sensitivity. The fructose sensitivity of *anac089*, L*er* and Col-0 was evaluated according to an earlier study by Li *et al.* (2011). We confirmed these results and showed that *anac060-1* was also highly sensitive to fructose (Supplementary Fig. S2A). For the swapping experiments, the transgenic seeds containing the p*ANAC060*::Cvi_*ANAC089* and p*ANAC089*::L*er*_*ANAC060* constructs in the *anac089* background together with the *anac089* mutant seeds and wild type L*er*, were germinated on 5.5%, 6%, and 6.5% fructose to assess their sugar sensitivity (Fig. 6B; Supplementary Fig. S2B). The percentage of healthy seedlings was examined based on the presence of green cotyledons. Only the line containing the p*ANAC089*::Cvi_*ANAC089* construct complemented the fructose sensitivity, confirming earlier results of Li *et al.* (2011). None of the swapping transformants revealed resistance to fructose (Fig. 6B). Both experiments indicate that neither the promotor nor the CDS of *ANAC089* and *ANAC060* can replace that of the other gene.

## Discussion

The NAC family of transcription factors is one of the largest plant-specific transcription factors containing over 100 genes that regulate multiple biological processes such as cell division, pathogen resistance and leaf senescence (Riechmann *et al.*, 2000; Kim *et al.*, 2006; Seo *et al.*, 2010; Yang *et al.*, 2011; Wu *et al.*, 2012). A comprehensive analysis was performed for the 75 NAC transcription factors in *Oryza sativa* (monocot) and 105 NAC family genes in Arabidopsis (dicot). Based on amino acid similarity, all the NAC family proteins were divided into two main groups and 18 sub-groups. *ANAC060*, *ANAC040*, and *ANAC089* were classified into the same sub-group, also referred to as OsNAC8 in *Oryza sativa* (Ooka *et al.*, 2003). In the Arabidopsis NAC protein classification, *ANAC060* and *ANAC089* belong to the same sub-family IIIa, whereas *ANAC040* is part of the Via sub-group (Zhu *et al.*, 2012). The phylogenetic tree revealed that *ANAC040* was retained from the At-beta duplication, and *ANAC060* and *ANAC089* were syntenic duplicates from the At-alpha duplication (Fig. 2B). Thus, *ANAC040* is likely more ancestral than *ANAC060* and *ANAC089*. NAC transcription factors are often pleiotropic; this is also the case for the genes studied here. *ANAC060* regulates seed dormancy and sugar (glucose and fructose) sensitivity (He *et al.*, 2014; Li *et al.*, 2014; Yu *et al.*, 2020). *ANAC040* inhibits seed germination under high salt concentrations, negatively regulates trichome formation and might play a role during dormancy cycling (Fig. 5B; Kim *et al.*, 2008; Tian *et al.*, 2017; Buijs *et al.*, 2020). *ANAC089* is reported as a central regulator of abiotic stress during seed germination and post-germinative development (Albertos *et al.*, 2021). Furthermore ANAC089 negatively regulates floral initiation and promotes seed germination under high concentrations of fructose (Li *et al*., 2010, 2011). Based on the high homology between *ANAC060*, *ANAC040*, and *ANAC089*, and the fact that these genes confer germination-related phenotypes, we aimed to study the possible redundancy in more detail.

### Promoter activity determines the functional diversity between *ANAC060* and *ANAC040*

*ANAC040* is a putative downstream target of ANAC060; this is supported by the reduced expression of *ANAC040* in *anac060-1* mutant seeds after 3 d of cold stratification (Fig. 4D). *ANAC060* and *ANAC040* both function in seed germination, and although both are expressed in seeds, their expression patterns are rather different. *ANAC060* expression levels are high in dry seeds, but significantly reduced during seed imbibition (Supplementary Fig. S5). We generated transgenic Arabidopsis seeds containing swaps of the nuclear localized forms of the ANAC060, ANAC089 and ANAC040 proteins driven by their respective promoters p*ANAC060,* p*ANAC089* or p*ANAC040* in *anac060-1*, *anac08*9, and *anac040* backgrounds, respectively. We have shown that the CDS of ANAC060 and ANAC040 are interchangeable, in the sense that the ANAC060 CDS can complement the *anac040* mutant salt sensitivity phenotype, and *vice versa*, the ANAC040 CDS complements the *anac060* mutant dormancy phenotype (Fig. 3). The lack of complementation of the dormancy phenotype using the *ANAC040* promoter is explained by the very low activity of this promoter in dry seeds (Fig. 4). Moreover, *ANAC060* expression was also highly induced by cold stratification (Supplementary Fig. S4); it may explain the complementation phenotype of p*ANAC060*::Col-*0*_*ANAC040ΔC* in the *anac040* background (Fig. 3F).

Promoter motif analyses were performed to reveal if the differences in promoter activity could be explained by the presence of known motifs. Several of the identified motifs have earlier been identified in the promoters of genes that are expressed in seeds, however most of them are stress-related. ABRE is a *cis*-acting element regulating ABA-related gene expression. This motif was initially detected in a wheat early-methionine-labelled (Em) gene, and recognized by a leucine zipper protein during seed maturation (Guiltinan *et al.*, 1990; Morris *et al.*, 1990). In Arabidopsis, it was indicated to be involved in regulating expression of *RD29* responding to drought and salt stresses (Narusaka *et al.*, 2003). MYB motifs have been indicated to play a role in the regulation the dehydration-responsive gene *RD22* (Abe *et al.*, 1997). The G-box motif determines seed-specific expression in tobacco transgenic plants (Ouwerkerk and Memelink, 1999). The GT1-motif has been suggested to activate transcription of light-dependent genes in tobacco transgenic plants (Lam and Chua, 1990). In p*ANAC060* four specific motifs were found, MYC, CAT-box, Activator Protein 1 (AP-1) and W-box. MYC motif with sequence CATGTG acts as a core DNA binding site in Arabidopsis *EARLY RESPONSIVE TO DEHYDRATION STRESS 1* (*ERD1*), which is a drought-induced gene (Tran *et al.*, 2004). The CAT-box is described to be related to meristem expression (Lescot *et al.*, 2002). AP-1 was initially identified to function in mice cell proliferation and survival, and has been described to function in response to oxidative stress in several other organisms (Karin *et al.*, 1997; Shaulian and Karin, 2001; Lev *et al.*, 2005; Scandalios 2005). The W-box motif is recognized by WRKY transcription factors (Rushton *et al.*, 2010), which are involved in biotic and abiotic stress, including seed dormancy and germination (Rushton *et al.*, 2012). The p*ANAC040* promoter contains two specific motifs; the stress response element (STRE) is able to regulate various stress-induced genes (Ruis and Schüller, 1995; Martínez-Pastor *et al.*, 1996; Estruch, 2000), and the GA-motif is a light responsive element (Lescot *et al.*, 2002; Table 1).

The *ANAC040* promoter was reported to be regulated by different environmental conditions, and might by induced by the binding of the TFs to the ABRE and STRE motifs (Table 1). Both the *ANAC040* transcript and protein levels were highly elevated during cold-imbibition, by 150 mM NaCl, and the combination of cold-imbibition and 150 mM NaCl, suggesting these three distinct conditions induce *ANAC040* in parallel or additively (Kim *et al.*, 2008). Based on this information, we tested the cold induced expression of *ANAC040* in the transgenic lines together with controls. *ANAC040* was clearly induced in the transgenic lines containing the pANAC040::Col-0_ANAC040ΔC and the pANAC040::Ler_ANAC060 transgenes, however the expression was significantly lower than in Col-*0* (Fig. 4D). The promotor sequence that was used for the cloning started 1722 bp upstream of the start codon, however the final 112 bp before the start codon was missing due to cloning difficulties. Motif analysis for the 112 missing nucleotides of the *ANAC040* promoter revealed that this region was enriched for the G-box and TATA-box motifs (Supplementary Fig. S6). The lack of these motifs might explain the lower expression of *ANAC040* in the in the transgenic lines containing the pANAC040::Col-0_ANAC040ΔC and the pANAC040::Ler_ANAC060 transgenes, however the expression was sufficient for the complementing the germination phenotype in salt. The random insertion of the construct might be another explanation for the lower expression.

Based on the fact that the p*ANAC060*::Col-0_*ANAC040ΔC* could overcome seed dormancy at 25 °C (Fig. 3B), we hypothesized that *ANAC040* could be involved in the regulation of dormancy cycling. Dormancy cycling occurs in imbibed seeds and might have occurred already before desiccation tolerance and primary seed dormancy existed. This hypothesis is supported by the fact the *ANAC040* is likely the ancestral gene from which *ANAC060* is derived (Fig. 2). *DOG1*, which is a main determinant of primary seed dormancy (Alonso-Blanco *et al.*, 2003; Bentsink *et al*., 2006, 2010), is also involved in the regulation of secondary seed dormancy, as was revealed from dormancy cycling induced by warm as well as cold stratification (Footitt *et al*., 2013, 2015; Murphey *et al.*, 2015). Based on our data, *ANAC060* and *ANAC040* have opposite functions compared with *DOG1*. *DOG1* is important for the induction of seed dormancy, whereas *ANAC060* and *ANAC040* inhibit seed dormancy (Fig. 3A, D). Notably, the expression pattern of *ANAC040* is also opposite to that of *DOG1* during dormancy cycling in the field (Fig. 5B). Moreover, the increased dormancy in the *anac060* mutants is likely not caused by increased *DOG1* expression, since *DOG1* transcript levels in mature seeds of the *anac060* mutants are similar to those in Col-*0* (Supplementary Fig. S5).

### Specific motifs in promoters of *ANAC060* and *ANAC040* might cause the distinct functions

Promoters are known as *cis*-regulatory sequences playing critical roles in regulating gene expression at the transcriptional level in all organisms (Wray *et al.*, 2003). There are various motifs in promoter sequences which are involved in regulation of genes (Halees *et al.*, 2003). The 12 overlapping motifs of the *ANAC060* and *ANAC040* promoters are shown in Table 1. Since these motifs are present in both promoters, it is not very likely that they determine the differences in expression of both genes. However, it should be noted that the frequency and location of these motifs in both promoters were not taken into account. In the promoter of *ANAC060*, four specific motifs are present, among them is the W-box (TTGACC) motif that is preferably bound by WRKY transcription factors (Ciolkowski *et al.*, 2008). The WRKY transcription factor family is indicated to be a key regulator in ABA-responsive signalling pathways, and several members of this family have been related to seed dormancy or germination as well (Rushton *et al.*, 2012). WRKY41 regulates seed dormancy by increasing *ABSCISIC ACID INSENSITIVE 3* expression (Ding *et al.*, 2014), WRKY6 is involved in ABA signalling by down-regulating *ETHYLENE RESPONSE DNA BINDING FACTOR 4* (*RAV1*; Huang *et al.*, 2016) and WRKY2 regulates seed germination and post-germination in response to ABA (Jiang and Yu, 2009). The presence of the W-box in the *ANAC060* promoter might indicate a role for WRKY transcription factors in the regulation of *ANAC060*. The STRE motif (AGGGG), that is found in the *ANAC040* promoter, is reported to be essential for the activation of transcription in response to stress (Ruis and Schüller, 1995; Martínez-Pastor *et al.*, 1996; Estruch, 2000; Table 1).

### Divergent functions of *ANAC060* and *ANAC089*

Genome-wide expression analysis shows that *ANAC089* is highly expressed in dry seeds (Winter *et al.*, 2007); its expression peaks at the same time as *ANAC060*. Nevertheless, the *ANAC089* promoter is not able to rescue the *anac060* dormancy phenotype when driving the *ANAC060* coding sequence, neither is the *ANAC060* promoter able to rescue the *anac089* phenotype when driving the *ANAC089* coding sequence. This might be explained by the different temporal and spatial expression patterns of *ANAC060* and *ANAC089*. Also, the coding sequence of *ANAC089* could not complement the *anac060* dormancy phenotype when driven by the *ANAC060* promoter, as was the case *vice versa* for the *ANAC060* coding sequence (Fig. 6). The sequence identity of both proteins is high, however the differences in function might be explained by structural differences. This was, for example, shown for *ANAC019*. Through X-ray crystallography, the ANAC019 NAC domain was identified to contain a twisted β-sheet surrounded by some helical elements instead of the helix-turn-helix motif that is common in several other NAC transcription factors; such a unique structural feature leads to diverse protein functions (Ernst *et al.*, 2004; Olsen *et al.*, 2005).

### The importance of ANAC060, ANAC040, and ANAC089 in regulating germination and early phases of seedling establishment

The highly homologous NAC transcription factors ANAC060, ANAC040 and ANAC089 regulate important transitions in the early phases of plant development. The timing of germination is crucial for successful establishment of a seedling in the environment, whether that is in an ecological or an agricultural setting. All three genes play a role in the interplay between the environment and the developmental switch that results in germination and/or seedling development. The natural genetic variation that is present for all three genes adds an interesting perspective to the regulation of these genes. Where the genetic variants that lack the TMD result in a constitutive expression of the gene, and thus in a gain-of-function phenotype, the membrane tethered allele has remained as well. This suggests an importance for the survival of the species, since the binding of the protein to the membrane results in an inhibition of germination (for *ANAC060*) and a sensitivity to either salt, fructose, abiotic stress (for *ANAC040* and *ANAC089*). For germination and seedling development to occur, the protein has to be released from the membrane, which for ANAC089 was shown to be directly affected by changes in the cellular redox status (Albertos *et al.*, 2021). Whether redox status has a similar effect on the translocation of the protein to the nucleus for ANAC060 and ANAC040, remains to be investigated. Insights into this allows the development of crop seeds for which germination and/or seedling development is insensitive to the environment; this will contribute to the development of climate stable crops. Due to the presence of natural variation this can be obtained by selective breeding; for this of course it remains to be investigated whether this natural genetic variation also occurs in crop species.

Through a transgenic swapping study, we showed that the genes encoding the NAC transcription factors *ANAC060* and *ANAC040*, but not *ANAC060* and *ANAC089* are functionally redundant. The phenotypic effects observed are caused by differences in the expression patterns of both genes, and these could be further investigated by replacing or knocking out specific motifs in each predictive promoter sequence, and examining the respective phenotypes of these alterations. To provide more evidence for a role of *ANAC040* in dormancy cycling, it would be useful to determine the dormancy cycling phenotype of the *anac040* mutant. However, because genetically modified organisms (GMO) are prohibited in field experiments in Europe, we will for now depend on the induction of secondary seed dormancy in laboratory conditions (Bouchaut and Asveld, 2020). So far, we have not been able to induce secondary dormancy in Col-0 and *anac040* mutant using the thermal treatment that was described by Footitt *et al.* (2017).

## Supplementary data

The following supplementary data are available at *JXB* online.

Table S1. List of primers used in Gateway cloning.

Table S2. Motif lists and the functional description of *ANAC060*, *ANAC040*, and *ANAC089*.

Table S3. List of 107 species used for synteny network analysis.

Fig. S1. Germination (%) of *anac040*, *anac060-1* mutants and Col-*0* in salt and mannitol treatments.

Fig. S2. Fructose sensitivity of the *anac060-1* and *anac089* mutants and transgenic lines.

Fig. S3. Swapping transgenic lines for *ANAC060* and its homologous genes.

Fig. S4. Relative *ANAC060* expression in Col-0 seeds under control condition and cold stratification.

Fig. S5. *DOG1* and *ANAC060* expression.

Fig. S6. Distribution of predicted motifs in the 112 missing nucleotides of *ANAC040* promoter.

erac232_suppl_Supplementary_Figures

erac232_suppl_Supplementary_Tables

## Data Availability

All data supporting the findings of this study are available within the paper and within its supplementary data published online.

## Abbreviations

MTFs: membrane bound transcription factors
NAM: no apical meristem
ATAF1, 2: *Arabidopsis thaliana ACTIVATING FACTOR1, 2*
CUC2: cup-shaped cotyledon (NAC)
TMD: transmembrane domain
