# Supplementary material for: The membrane associated NAC transcription factors ANAC060 and ANAC040 are functionally redundant in the inhibition of seed dormancy in Arabidopsis thaliana
Source: J Exp Bot. 2022 May 23;73(16):5514–28. doi: 10.1093/jxb/erac232 (PMC9467645; doi:10.1093/jxb/erac232)
Supplement: erac232_suppl_Supplementary_Figures [file erac232_suppl_supplementary_figures.pdf]

Supplementary figures

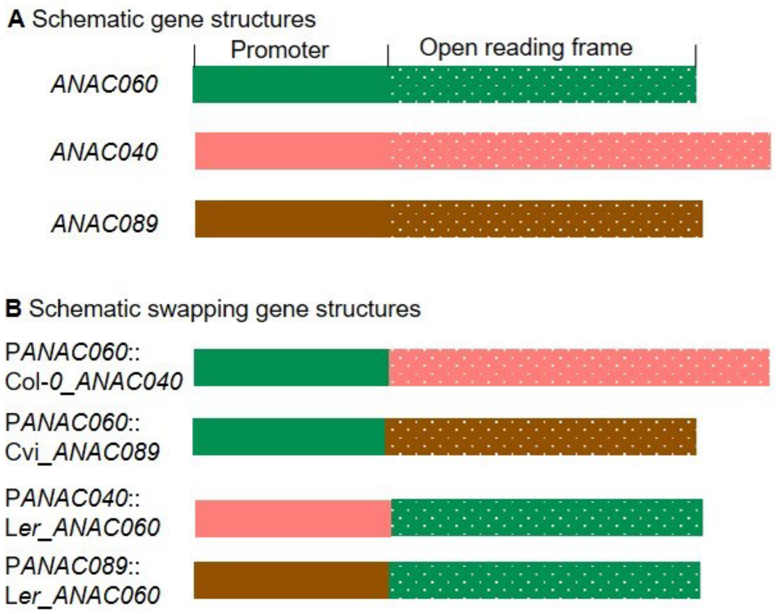

Supplementary Figure 1. Swapping transgenic lines for *ANAC060* and its homologous genes. Transgenic lines were made by switching promoters and genomic CDSs of *ANAC060*, *ANAC040* and *ANAC089* (A), the recombined constructs were illustrated in (B) and transformed into respective mutants.

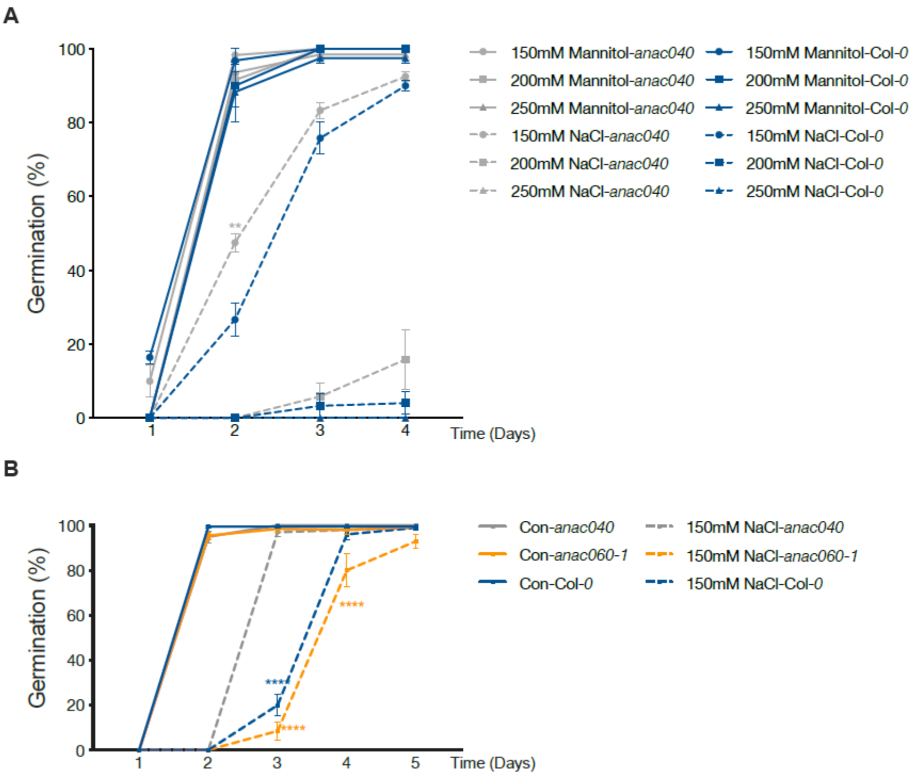

Supplementary Figure 2. Germination dose response curves in different concentrations of mannitol and NaCl. (A). Germination percentages of *anac040* and Col-*0* in different salt and mannitol concentrations. (B). Single mutants *anac040*, *anac060-1* and Col-*0* in water (Con) and 150mM NaCl treatment; Mean values of three replicates and the standard errors are indicated. Statistical significances were calculated two-way ANOVA. Asterisks indicate significant differences in germination percentage between the *anac040* mutant and Col-*0* at the given time-point and treatment in (A) and differences in germination percentage for *anac060-1* and Col-*0* when compared to *anac040* mutant at the given time-point and treatment in (B). (**: $P \leq 0.01$ ****: $P \leq 0.0001$).

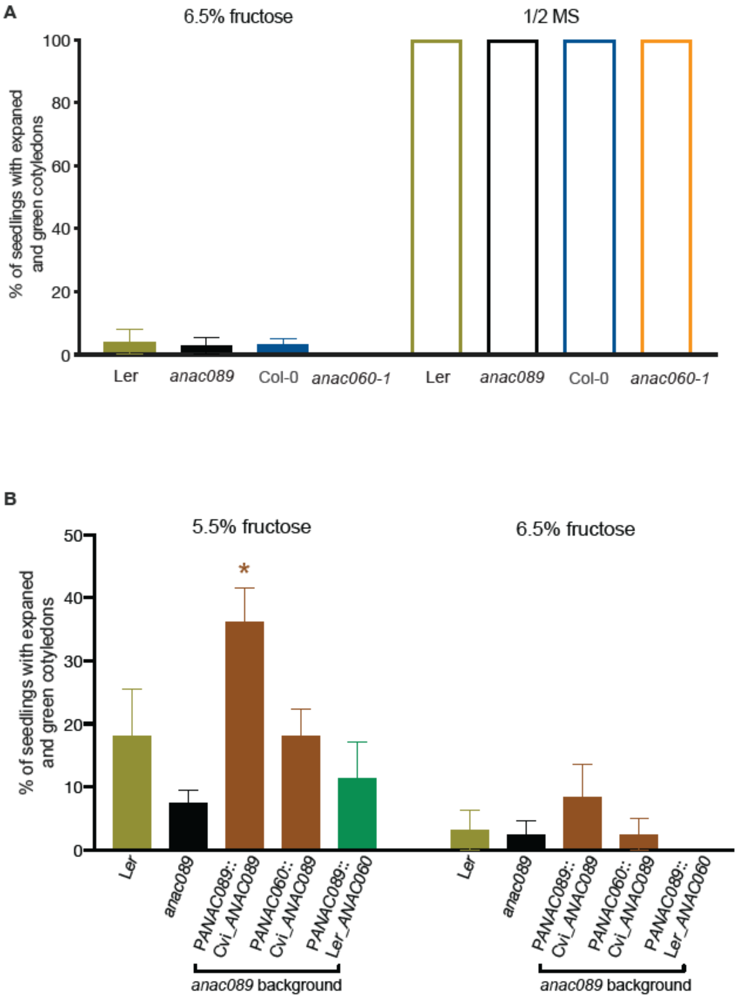

Supplementary Figure 3. Fructose sensitivity measured by seedling greening. (A) Seedling greening of the single mutants *anac060-1* and *anac089* and their respective wildtypes on ½ MS medium and ½ MS plates containing 6.5% fructose. (B). Seedling greening for L*er*, *anac089* and transgenic lines on ½ MS medium plates containing 5.5% and 6.5% fructose. Statistical significances were calculated using one-way ANOVA. Bars indicate the mean value of three replicates and the standard error. Asterisks indicate significant differences between *anac089* and respective transgenic lines (*: $P \leq 0.05$).

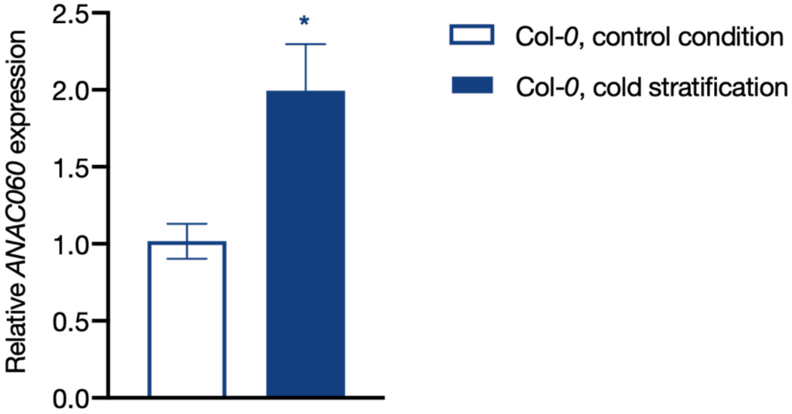

Supplementary Figure 4. Relative *ANAC060* expression in Col-*0* seeds in control conditions and after cold stratification. Col-*0* seeds were after-ripened and further imbibed for 6 hours under control (ambient) condition and 4°C (cold stratification). Statistical significances were calculated using Paired t test. Bars indicate the mean value of four replicates and the standard error. Asterisks indicate significant differences between control condition and cold stratification (*: P ⩽ 0.05)

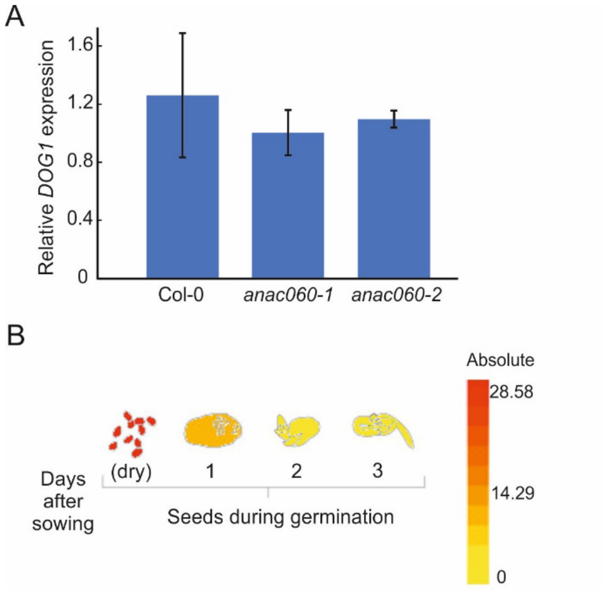

Supplementary Figure 5. A. Relative *DOG1* expression in mature dry seeds of Col-0 and the *anac060-1* and *anac060-2* mutants. Bars indicate the mean value of three replicates and the standard error. B. *ANAC060* expression in dry and imbibed seeds. Data as presented at the Arabidopsis eFP browser (http://bar.utoronto.ca/efp/cgi-bin/efpWeb.cgi) Klepikova Atlas (Klepikova et al., 2016). Color codes indicate the level of expression.

Reference:

Klepikova AV, Kasianov AS, Gerasimov ES, Logacheva MD, & Penin AA. 2016. A high resolution map of the Arabidopsis thaliana developmental transcriptome based on RNA‐seq profiling. The Plant Journal 88, 1058-1070.

A

```
+ TCTGAAGCCG CCAAATATCA AAAAACACAA AACCTTTACC CGAAGCAAAG TAGCTGCACA CTTGCCATTT
- AGACTTCGGC GGTTTATAGT TTTTTGTGTT TTGGAAATGG GCTTCGTTTC ATCGACGTGT GAACGGTAAA

+ ATAAGGCATG TTTCTTCATC TAAAGCAAAG TATTTTCGAG TTCCTGAGCC AAAAAGATCA AACA
- TATTCCGTAC AAAGAAGTAG ATTTCGTTTC ATAAAAGCTC AAGGACTCGG TTTTTCTAGT TTGT
```

B

```
+ TCTGAAGCCG CCAAATATCA AAAAACACAA AACCTTTACC CGAAGCAAAG TAGCTGCACA CTTGCCATTT
- AGACTTCGGC GGTTTATAGT TTTTTGTGTT TTGGAAATGG GCTTCGTTTC ATCGACGTGT GAACGGTAAA

+ ATAAGGCATG TTTCTTCATC TAAAGCAAAG TATTTTCGAG TTCCTGAGCC AAAAAGATCA AACA
- TATTCCGTAC AAAGAAGTAG ATTTCGTTTC ATAAAAGCTC AAGGACTCGG TTTTTCTAGT TTGT
```

Supplementary Figure 6. Distribution of predicted motifs in the 112 missing nucleotides of ANAC040 promoter. In panel A, B two times the same sequence is indicated. (A) The missing part is starting from 'AAACACAA' after orange highlighted box and lasts until the end of the sequence. The darker yellow block indicates the G-box. (B) The blue box indicates the TATA-box . Analysis was performed using PlantCARE (Lescot *et al*., 2002).
